# Context-Responsive Anticoagulation Reduces Complications in Pediatric Extracorporeal Membrane Oxygenation

**DOI:** 10.3389/fcvm.2021.637106

**Published:** 2021-06-10

**Authors:** John C. Lin, Lauren M. Barron, Adam M. Vogel, Ryan M. Colvin, Sirine A. Baltagi, Allan Doctor, Avihu Z. Gazit, Mary Mehegan, Nicole O'Connor, Ahmed S. Said, Mark Shepard, Michael Wallendorf, Philip C. Spinella

**Affiliations:** ^1^Division of Pediatric Critical Care Medicine, Department of Pediatrics, Washington University School of Medicine, St. Louis, MO, United States; ^2^Division of Pediatric Surgery, Department of Surgery, Washington University School of Medicine in St. Louis, St. Louis, MO, United States; ^3^Pediatric Computing Facilities, Department of Pediatrics, Washington University School of Medicine, St. Louis, MO, United States; ^4^St. Louis Children's Hospital, St. Louis, MO, United States; ^5^Division of Biostatistics, Washington University School of Medicine, St. Louis, MO, United States

**Keywords:** pediatric, extracorporeal support, anticoagulation, transfusion, hemorrhagic complications

## Abstract

**Purpose:** We sought to determine the impact of a comprehensive, context-responsive anticoagulation and transfusion guideline on bleeding and thrombotic complication rates and blood product utilization during extracorporeal membrane oxygenation (ECMO).

**Design:** Single-center, observational pre- and post-implementation cohort study.

**Setting:** Academic pediatric hospital.

**Patients:** Patients in the PICU, CICU, and NICU receiving ECMO support.

**Interventions:** Program-wide implementation of a context-responsive anticoagulation and transfusion guideline.

**Measurements:** Pre-implementation subjects consisted of all patients receiving ECMO between January 1 and December 31, 2012, and underwent retrospective chart review. Post-implementation subjects consisted of all ECMO patients between September 1, 2013, and December 31, 2014, and underwent prospective data collection. Data collection included standard demographic and admission data, ECMO technical specifications, non-ECMO therapies, coagulation parameters, and blood product administration. A novel grading scale was used to define hemorrhagic complications (major, intermediate, and minor) and major thromboembolic complications.

**Main Results:** Seventy-six ECMO patients were identified: 31 during the pre-implementation period and 45 in the post-implementation period. The overall observed mortality was 33% with no difference between groups. Compared to pre-implementation, the post-implementation group experienced fewer major hemorrhagic and major thrombotic complications and less severe hemorrhagic complications and received less RBC transfusion volume per kg.

**Conclusions:** Use of a context-responsive anticoagulation and transfusion guideline was associated with a reduction in hemorrhagic and thrombotic complications and reduced RBC transfusion requirements. Further evaluation of guideline content, compliance, performance, and sustainability is needed.

## Introduction

Extracorporeal membrane oxygenation (ECMO) is a vital, life-sustaining therapy for the management of patients with refractory respiratory or cardiac failure. To enable extracorporeal blood flow and prevent thrombotic and embolic complications, systemic anticoagulation is mandatory (1). Resulting bleeding and thrombotic complications are a major consequence of ECMO therapy and carry significant mortality and morbidity (2, 3). In the absence of an evidence-based approach to anticoagulation and transfusion management, wide clinical variability exists among ECMO centers (4). Mitigating risk and managing development of bleeding and clotting complications is of paramount importance and represents one of the major challenges facing ECMO management.

Systemic anticoagulation in ECMO is most commonly accomplished by unfractionated heparin infusion. Heparin dose has traditionally been titrated targeting various preset ranges for activated clotting time (ACT) (1, 5, 6). Although widely used to monitor systemic anticoagulation, ACT correlates poorly with both heparin pharmacokinetics and pharmacodynamics (7–9). Alternatively, activated partial thromboplastin time (aPTT), anti-factor Xa levels, antithrombin III (AT-III) activity, fibrinogen levels, thromboelastography (TEG) with and without heparinase, and platelet mapping have been used in varying combinations with or without ACT to guide anticoagulation and transfusion management (7–12). ECMO centers use institution-specific testing to guide anticoagulation and transfusion management without direct evidence of this approach on outcomes (4).

In addition to pharmacologic therapy, blood product transfusion therapy also impacts coagulation status. The Extracorporeal Life Support Organization (ELSO) has published transfusion guidelines (6) that have not been validated for clinical efficacy in neonatal and pediatric ECMO patients (13, 14). As with anticoagulation management, in the absence of definitive evidence, transfusion thresholds vary greatly among ECMO centers (4).

The role that patient clinical context plays on hemostasis has not been previously addressed. The patient's underlying disease contributes to derangements in hemostasis (1, 15), and the introduction of the interaction between blood and ECMO circuit foreign surfaces further disturbs the hemostatic balance (16). In response, we developed an institutional guideline for anticoagulation and transfusion therapy based on the limited ECMO and broader critical illness literature as well as local and international expert opinion and experience. The driving premise of these guidelines was that the patient's clinical status and evidence of bleeding or clotting, not preset thresholds, should dictate anticoagulation and transfusion goals. Similarly, comprehensive laboratory testing rather than a single result allows better understanding of the complicated interactions among patient, illness, and artificial circuit. This guideline was implemented in all intensive care units at Saint Louis Children's Hospital: pediatric, cardiovascular, and neonatal, hereafter referred to collectively as ICU. The purpose of this study was to determine the clinical impact of the newly developed comprehensive, context-responsive anticoagulation and transfusion guideline (hereafter, referred to as “Guideline”). We hypothesized that bleeding and thrombotic complications in children receiving ECMO would decrease following Guideline implementation.

Preliminary study results have been previously reported as an abstract (17).

## Materials and Methods

The new Guideline assigned primary importance to the clinical assessment of the hemorrhagic and clotting risk and current coagulation status and started with patient assignment into one of three clinical states: No clotting/No bleeding, Clotting, or Bleeding (Supplementary Material), each with a customized approach to monitoring and therapy. All post-operative patients were assigned to a specific clinical state based on discussion among the medical and surgical teams and determination of specific initial platelet and ACT goals. After designation of a clinical state and assignment to the correlating Guideline pathway, a comprehensive laboratory assessment provided target ranges or thresholds for anticoagulation management and transfusion that were specific to the assigned clinical state. This laboratory schedule included complete blood count (CBC), ACT, prothrombin time (PT), international normalized ratio (INR), activated partial thromboplastin time (aPTT), anti-factor Xa activity (anti-Xa), thromboelastography (TEG) with and without heparinase, and platelet mapping. The entire Guideline along with flowcharts is available in the Supplementary Material.

Official implementation of the Guideline occurred in August 2013 and was preceded by comprehensive education over several months in each ICU. This education component included tailored sessions for each group of the interprofessional ICU and ECMO teams. The pre-implementation period was thus defined as January 1 to December 31, 2012, to avoid practice change based on these education sessions prior to official implementation. The post-implementation period was defined as September 1, 2013, to December 31, 2014. All patients <18 years of age at the time of ECMO initiation were included in both periods. Patients cannulated at another institution and subsequently transferred to our hospital were excluded. Prolonged ECMO ≥28 days is required for refractory cardiac or respiratory failure; happens infrequently; and is associated with a very different complication, morbidity, and mortality rate (18). Patients requiring prolonged ECMO for ≥28 days were thus excluded.

Data for the pre-implementation cohort was collected retrospectively via chart review. The post-implementation cohort data collection was planned prospectively and consisted of only clinical data that was already being recorded as part of routine clinical care. The Washington University School of Medicine Institutional Review Board approved this study with waiver of consent.

### Data Collection

Standard demographic data was collected and included age, gender, ethnicity, gestational age if <31 days old on the day of cannulation, weight, and height. Primary and secondary diagnoses resulting in the need for ECMO were recorded. Presence or absence of the following preexisting conditions or active clinical problems prior to ECMO initiation was noted: echocardiography findings consistent with pulmonary hypertension, malignancy, chromosomal disorders, neurological disorders or deficits, trauma, and documented infection. Pre-ECMO therapies were noted and included the use of inhaled nitric oxide, inhaled prostacyclin, and renal replacement therapy (RRT). The recorded ECMO technical details included mode of cannulation, site(s) of cannulation, type of pump, primary diagnostic classification (neonatal or pediatric; respiratory or cardiac), and need for extracorporeal cardiopulmonary resuscitation (ECPR). Blood product usage, including type of product and total daily amount, was recorded for all transfused blood products.

Clinical outcome measures collected included length of hospital admission, length of ICU admission, duration of intubation, and survival to hospital discharge. Acute kidney injury was defined as a creatinine greater than twice the baseline at cannulation. Infectious complications were defined as antibiotic use for indications other than prophylaxis or documented symptoms of new infection, confirmed by culture and initiation of new antibiotic coverage. Intracranial hemorrhage (ICH) was defined as a positive finding reported on neuroimaging ordered at the discretion of the primary team based on clinical indications.

### Coagulation Complications

Coagulation complications were categorized according to a novel grading system that stratified type (hemorrhagic vs. thrombotic) and degree (major vs. intermediate vs. minor) (Table 1). Complication category was determined and assigned by LMB with review and confirmation by AMV. This was accomplished by chart review in the retrospective arm and by real-time bedside documentation by the ECMO specialist in the prospective arm. Hemorrhagic complications occurring <24 h after the previous hemorrhagic complication were counted separately if a new source of hemorrhage was documented or there were ongoing RBC transfusion requirements. For thromboembolic events, circuit interventions <48 h apart, which required replacement of circuit components, were counted as a single event unless the same component was replaced twice.

**Table 1:** Bleeding and thrombotic complication categories and examples.

**Hemorrhagic—major**	Bleeding requiring surgical intervention Examples: - Wound exploration - Laparotomy - Thoracotomy
	Bleeding requiring discontinuation of ECLS and decannulation Example: grade IV intraventricular hemorrhage
	Bleeding causing death
	Bleeding requiring > 20 ml/kg/day RBC transfusion
**Hemorrhagic—intermediate**	Neurologic complication requiring pharmacologic^a^ adjustments but not requiring decannulation Example: new grade I intraventricular hemorrhage
	Other internal or external bleeding complication requiring pharmacologic and additional interventions Examples: - Pulmonary bleeding requiring heparin adjustment and a change in ventilator parameters - Gastrointestinal bleeding requiring heparin adjustment and holding enteral feeds - Urologic bleeding requiring heparin adjustment and removing the Foley catheter
**Hemorrhagic—minor**	Bleeding requiring 10–20 ml/kg/day RBC transfusion
	Bleeding requiring ONLY pharmacologic intervention^a^ or non-surgical intervention Examples: application of topical hemostatic agent to skin incision
**Thromboembolic—major**	Patient thrombotic/embolic event requiring discontinuation of ECLS or resulting in patient death Acute, unexpected circuit thrombosis requiring component or circuit change
**Thromboembolic—minor**	Clinically overt patient or circuit thromboembolic event requiring pharmacologic intervention Examples: increasing heparin dose

a *Includes heparin adjustments or transfusing coagulation factor such as FFP or cryoprecipitate*.

### Statistical Analysis

Unadjusted comparisons of baseline patient characteristics, complications, and blood product utilization where conducted using Fisher's exact test for nominal variables or Wilcoxon rank-sum test for continuous variables. Multiple logistic regression models were developed to evaluate the association of Guideline implementation with hemorrhagic or thrombotic complications. Potential confounder candidates included age, ethnicity, gender, pulmonary hypertension diagnosis, chromosomal disorder, neurologic disorder, inhaled prostacyclin use, pre-ECMO renal replacement therapy, eCPR, ECMO diagnosis class (respiratory vs. cardiac), ECMO mode, and pump type. Using a *p*-value threshold of 0.20 for inclusion in the final model, each candidate variable was assessed in stepwise logistic regression models to identify independent association with the outcomes of hemorrhagic or thrombotic complications (19). Analysis was completed with SAS v. 9.4 (Cary, NC) statistical software.

## Results

A total of 76 subjects were enrolled. In the 36 patients identified for the pre-implementation cohort, two patients were > 18 years old and three had incomplete data, leaving 31 subjects. In the 52 patients identified for the post-implementation cohort, two patients were > 18 years old, three had incomplete data, and two required ECMO courses exceeding 28 days, leaving 45 subjects. The pre- and post-implementation groups were well matched with respect to demographics and primary indication for ECMO (neonatal respiratory, neonatal cardiac, pediatric respiratory, and pediatric cardiac) (Table 2). We observed an overall mortality of 25/71 (33%) with no difference in mortality between cohorts. The groups also had similar ICU- and ventilator-free days and similar rates of acute kidney injury (Table 3).

**Table 2:** Patient characteristics prior to ECMO cannulation.

**Demographic**	**Pre-implementation *N* = 31**	**Post-implementation *N* = 45**	***p*^a^**
Ethnicity			0.58
Black	5 (16.1)	11 (24.4)	
White	23 (74.2)	27 (60.0)	
Hispanic	1 (3.2)	1 (2.2)	
Other	2 (6.5)	6 (13.3)	
Gender			0.49
Male	16 (51.6)	19 (42.2)	
Age (years)	0.33 (0.03, 4)	0.33 (0.02, 8)	0.76
Neonate	10 (32.3)	20 (44.4)	0.34
Diagnosis class			0.15
Respiratory	9 (29.0)	21 (46.7)	
Cardiac	22 (71.0)	24 (53.3)	
Inhaled NO or prostacyclin	14 (45.2)	18 (40.0)	0.81
Pulmonary hypertension	11 (37.9)	19 (42.2)	0.81
Malignancy	0 (0.0)	0 (0.0)	–
Chromosomal disorder	7 (22.6)	5 (11.1)	0.21
Inborn error in metabolism	0 (0.0)	0 (0.0)	–
Neuro disorder	4 (12.9)	9 (20.5)	0.54
Trauma	0 (0.0)	0 (0.0)	–
ECPR	9 (29.0)	11 (24.4)	0.79
Pre ECMO RRT	1 (3.2)	1 (2.2)	1.00
ECMO configuration			0.10
VA	28 (90.3)	32 (71.1)	
VVDL	3 (9.7)	12 (26.7)	
VVDL+V	0 (0.0)	1 (2.2)	
Pump			0.34
Roller	22 (71.0)	26 (59.1)	
Centrifugal	9 (29.0)	18 (40.9)	

*Data presented as n (%) or median (25, 75th%tile)*.

*ECPR, extracorporeal cardiopulmonary resuscitation; NO, nitric oxide; ECMO, extracorporeal membrane oxygenation; RRT, renal replacement therapy; VA, venoarterial; VV, venovenous; DL, dual lumen*.

a *Fisher's exact test or Wilcoxon rank-sum test*.

**Table 3:** Outcomes and complications.

**Outcome**	**Total**	**Pre-implementation**	**Post-implementation**	***p*^a^**
	**(*N* = 76)**	**(*N* = 31)**	**(*N* = 45)**	
Mortality (any cause)	25 (32.9)	13 (41.9)	12 (26.7)	0.22
Average run (days)	6 (4, 8)	5 (3, 10)	6 (4, 8)	1.00
28-day ICU-free days^b^	0 (0, 9)	0 (0, 8)	0 (0, 10)	1.00
60-day ICU-free days^c^	11 (0, 41)	15 (0, 40)	9 (0, 42)	0.92
28-day ventilator-free days^b^	0 (0, 21)	4 (0, 20)	0 (0, 22)	0.96
60-day ventilator-free days^c^	31 (0, 53)	36 (0, 52)	31 (0, 54)	0.76
Intracranial hemorrhage	14 (18.4)	6 (19.4)	8 (17.8)	1.00
Acute kidney injury	13 (17.1)	7 (22.6)	6 (13.3)	0.36
Most severe hemorrhagic complication^d^				0.006
Major	22 (29.0)	14 (45.2)	8 (17.8)	
Major or intermediate	29 (38.2)	16 (51.6)	13 (28.9)	
Any	38 (50.0)	16 (51.6)	22 (48.9)	
Major thromboembolic	7 (9.2)	6 (19.4)	1 (2.2)	0.02

*Data presented as n (%) or median (25, 75th%tile)*.

a *Fisher's exact test or Wilcoxon rank-sum test*.

b *In first 28 days after ECMO decannulation. Patients who died were assigned a value of 0*.

c *In first 60 days after ECMO decannulation. Patients who died were assigned a value of 0*.

d *See Figure 1*.

### Hemorrhagic and Thrombotic/Embolic Complications

**Figure 1:**
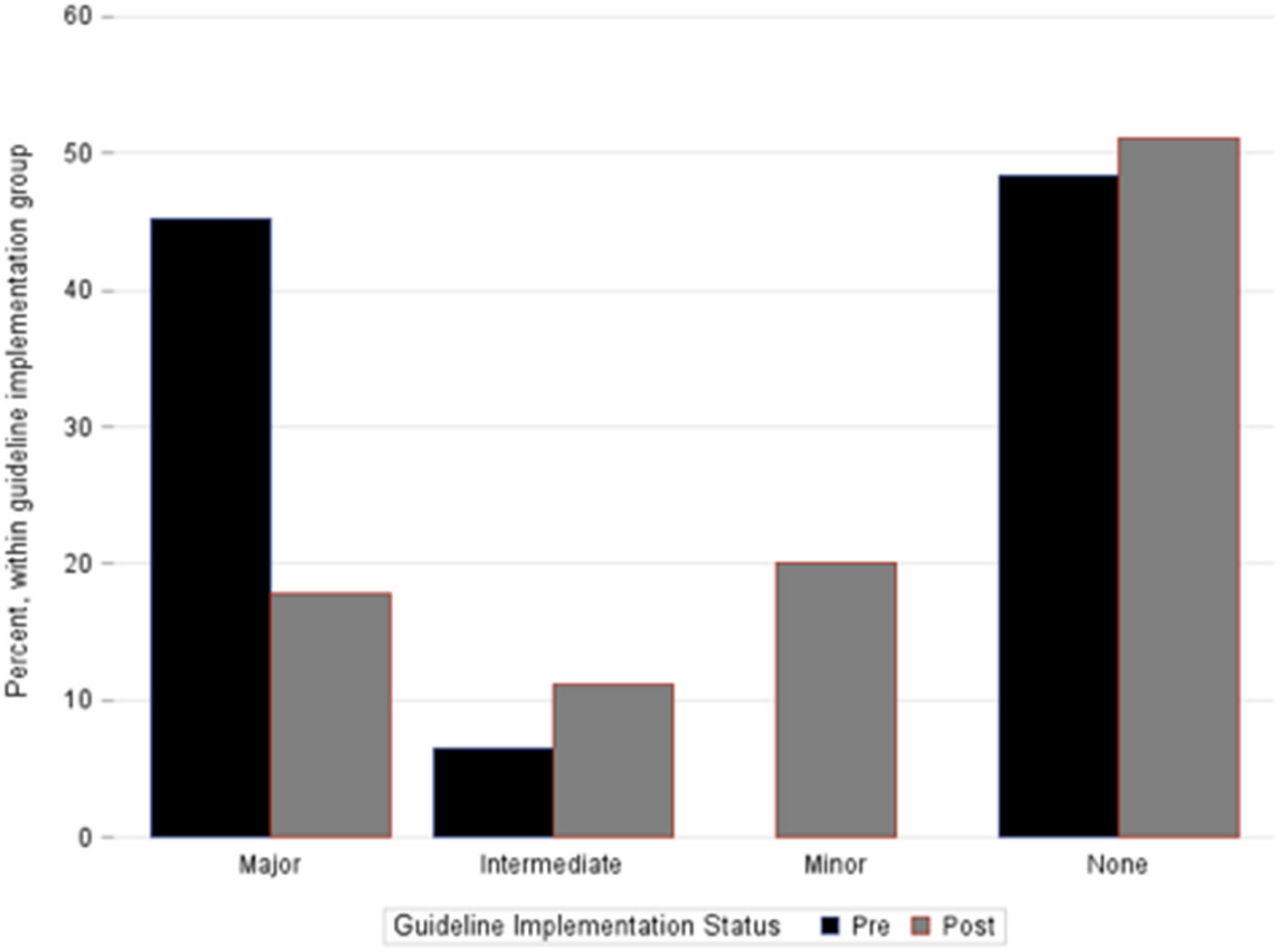
Percentage of patients within each bleeding category severity cohort. Bar groupings represent the most severe hemorrhagic complication each individual patient suffered during the ECMO course. No patients in the pre-implementation cohort experienced only a minor hemorrhagic complication. Fisher's exact test, *p* = 0.006.

The total number of patients experiencing any degree of hemorrhagic complication was not different between cohorts. However, compared to pre-implementation, the post-implementation cohort had fewer major hemorrhagic complications (18 vs. 45%, *p* = 0.02), a trend toward fewer major or intermediate hemorrhagic complications (29 vs. 52%, *p* = 0.06), and fewer thromboembolic complications [2 vs. 19%, *p* = 0.02 (Table 3)]. In patients who experienced any hemorrhagic complications, those in the post-implementation group experienced a lower maximal severity of bleeding (Table 3 and Figure 1). Fourteen patients overall experienced an ICH. There was no difference in occurrence of ICH between pre- and post-implementation groups (Table 3). Irrespective of Guideline implementation status, of the 14 patients who experienced an ICH, eight died (57%) compared to 17 deaths in the 62 patients without ICH (28%), *p* = 0.06. Irrespective of Guideline implementation status, treatment with either VA- or VV-ECMO was not associated with hemorrhagic complications: major (19/60, 32 vs. 3/16, 19%, *p* = 0.37), major + intermediate (23/60, 38 vs. 6/19, 32%, *p* = 1.00). Similarly, diagnosis category of respiratory vs. cardiac ECMO was not associated with hemorrhagic complications: major (8/30, 27 vs. 14/46, 30%, *p*-0.8), major + intermediate (11/30, 37 vs. 18/46, 39%, *p* = 1.0).

Baseline characteristics listed in Table 2 were analyzed using multivariable stepwise regression (*p*-value ≤ 0.20 for inclusion) to assess potential confounding of the relationship between Guideline implementation status and hemorrhagic complications. Because there were so few thrombotic complications, this regression analysis was not performed. The union of candidate variables from two models for (1) major or (2) major + intermediate hemorrhagic complications yielded four variables for inclusion: neonatal status, presence of chromosomal disorder, ECMO pump type, and guideline status. In these models, Guideline implementation was associated independently with decreased occurrence of major hemorrhagic complication (OR 0.20, CI 0.06–0.68, *p* = 0.01) and trended toward significance with fewer major or intermediate hemorrhagic complications (OR 0.41, CI 0.15–1.14, *p* = 0.09) (Table 4).

**Table 4:** Adjusted logistic regression models.

**Characteristics compared^a^**	**Model 1**		**Model 2**	
	**OR (95% CI)**	***p***	**OR (95% CI)**	***p***
Cohort, post- vs. pre-implementation	0.20 (0.06–0.68)	0.01	0.41 (0.15–1.14)	0.09
Neonate, *y* vs. *n*	0.86 (0.20–3.69)	0.84	0.43 (0.12–1.52)	0.19
Chromosomal disorder, *y* vs. *n*	1.95 (0.47–8.16)	0.36	1.39 (0.37–5.27)	0.63
Pump, centrifugal vs. roller	4.51 (1.05–19.32)	0.04	1.42 (0.41–4.95)	0.58

*Model 1: dependent variable: MAJOR hemorrhagic complication. ROC AUC = 0.74*.

*Model 2: dependent variable: MAJOR or INTERMEDIATE hemorrhagic complication. ROC AUC = 0.67*.

a *The following additional variables in Table 2 were evaluated but did not meet the statistical inclusion criteria threshold of p ≤ 0.2: ethnicity, gender, pulmonary hypertension diagnosis, neurologic disorder, inhaled prostacyclin use, pre-ECMO renal replacement therapy, eCPR, ECMO diagnosis class (respiratory vs. cardiac), and ECMO mode*.

### Transfusions

Prior to Guideline implementation, clinicians transfused blood products at their discretion. The Guidelines provided specific transfusion criteria and thresholds for all blood products. Similar numbers of patients in both cohorts received RBC, platelets, fresh frozen plasma (FFP), or cryoprecipitate. However, the total RBC volume transfused was lower in the post-implementation group: 72 vs. 46 mL/kg, *p* = 0.02. There were no significant differences in transfusion volumes in the other products (Table 5).

**Table 5:** Blood product transfusions pre- and post-implementation.

**Blood product**	**Pre-implementation**	**Post-implementation**	***p*^a^**
	**(*N* = 31)**	**(*N* = 45)**	
Red blood cells (RBC)			
Patients transfused	30 (96.8)	42 (93.3)	0.64
Total amount transfused per patient, mL/kg	72 (38, 150)	46 (18, 76)	0.02
Platelets			
Patients transfused	27 (87.1)	41 (91.1)	0.71
Total amount transfused per patient, mL/kg	35 (13, 100)	35 (13, 76)	0.69
Fresh frozen plasma (FFP)			
Patients transfused	23 (74.2)	29 (64.4)	0.46
Total amount transfused per patient, mL/kg	20 (0, 38)	10 (0, 22)	0.06
Cryoprecipitate			
Patients transfused	16 (51.6)	28 (62.2)	0.48
Total amount transfused per patient, mL/kg	1 (0, 5)	2 (0, 6)	0.42

*Data presented as n (%) or median (25, 75th%tile)*.

a *Fisher's exact test or Wilcoxon rank-sum test*.

## Discussion

Following implementation of a comprehensive, context-responsive anticoagulation and transfusion guideline, we observed a significant decrease in the number of hemorrhagic and thrombotic complications and the severity of hemorrhagic complications. We also observed a reduction in total RBC transfusion volume per patient without changes in non-RBC blood product utilization. To our knowledge, this is the first report of an anticoagulation and transfusion guideline that explicitly defines different target ranges and thresholds for pharmacologic anticoagulation titration and blood product administration based on the clinical context and a comprehensive panel of laboratory testing.

We believe that these results provide preliminary evidence supporting proof of concept of the primary principles underlying these Guidelines. First, anticoagulation and transfusion management in ECMO should begin with an assessment of the patient's clinical status and should not be driven solely by laboratory-based thresholds. Second, to guide coagulation management, a panel of laboratory testing provides a more comprehensive assessment than any individual test. Subsequent decision-making then relies on a lab testing strategy guided by the patient's clinical condition and hemostatic state. Third, we propose a categorization system to better quantify and standardize the classification of hemorrhagic and clotting complications to allow for objective comparison of these events over time. Lastly, we explicitly link blood product transfusion criteria with context-specific transfusion ranges based on the patient's assigned clinical state.

In the latest edition of the Red Book, 5th edition, ELSO recommends heparin titration to achieve an anti-Xa range of 0.3 to 0.7 and provides the following transfusion parameters: hematocrit of 30%, platelets of 80,000 to 100,000 per μL, INR of < 2, and fibrinogen of 100–150 mg/dL for RBC, platelets, FFP, and cryoprecipitate, respectively (16). However, in an attempt to limit blood product exposure and reduce patient bleeding events, individual centers have targeted a lower heparin effect (20), RBC transfusion thresholds as low as a hematocrit of 24% (20), platelet transfusion thresholds as low as 40,000 per μL (20), and INR as low as 1.5 (21). These specific guidelines attempt to account for heparin pharmacokinetics (anti-Xa) and pharmacodynamics (e.g., ACT, INR, TEG). However, heparin pharmacokinetics and pharmacodynamics are markedly altered in varying directions and degrees based on the cause of critical illness (1, 15, 22) and from the introduction of the ECMO circuit–blood interface (16).

The variation in clinical practice and wide range in reported experience and approach underscores the complexity of anticoagulation and transfusion management in ECMO. The etiology of bleeding and thrombotic complications in ECMO patients is multifactorial and not only due to anticoagulant medication effect or blood product administration. Underlying disease states further derange hemostatic status, and the introduction of the interactions between blood and ECMO circuit artificial surfaces further complicate attempts to “control” anticoagulation. Adding to this complexity, no single laboratory test has been demonstrated to be the best predictor of thrombotic or bleeding complications during systemic anticoagulation during ECMO support (8, 9, 23), leading to significant variability in what laboratory tests are obtained to guide ECMO anticoagulation management (4).

Recently, Northrop et al. at Vanderbilt University demonstrated that standardization of an anticoagulation lab monitoring protocol alone resulted in decreased blood product utilization, fewer hemorrhagic complications, and prolonged circuit life (24). The authors did not have sufficient patient numbers to determine whether or not survival was impacted. As our Guidelines did, the Vanderbilt group incorporated clinical evidence of bleeding or clotting into the scheduled frequency and type of lab testing performed. However, unlike our Guidelines, the Vanderbilt laboratory monitoring algorithm did not alter guidance on transfusion or anticoagulation management based on clinical context, in addition to lab results. Rather, laboratory value ranges were targeted as indications for heparin titration or blood product transfusion, irrespective of the patient's clinical context or the clinically observed presence of bleeding, clotting, both, or neither observed in the patient or the ECMO circuit.

In the accompanying editorial to the Vanderbilt report, Lequier and Massicotte highlight the lack of evidence and argue that current practice actually optimizes survival and outcomes: “The lack of evidence for current therapies in … ECMO is pervasive especially for the specifics of anticoagulation management of these patients” (25). Contributing to this evidence gap has likely been the historical “one size fits all” approach to coagulation monitoring and intervention and transfusion strategies. Such an approach lacks the flexibility needed with different clinical contexts between patients or changing conditions in a single patient over time.

Beginning with a novel scoring system that graded bleeding and clotting severity, our Guidelines emphasized the need to reach consensus upon the patient's clinical status as one of three states: no bleeding/clotting vs. bleeding vs. clotting. Once the clinical condition was assigned, the Guidelines then provided recommendations for laboratory testing frequency and therapeutic interventions with different ranges and goals that were based on the combination of clinical state and lab monitoring. By adjusting both lab testing frequency and intervention thresholds for specific test results, we observed less RBC transfusion volume, as others have reported (24). More importantly, in multivariable analyses, we also observed a decrease in the number and severity of hemorrhagic complications as well as the number of thromboembolic complications.

There were several limitations of the study. The small cohort sizes are an important one, reflecting the preliminary nature of these findings from our single center. Given the relatively small patient numbers, in order to avoid multiple comparisons, we did not perform repeated statistical analyses of each individual subcategory that comprised hemorrhagic complication severity. Similarly, we also did not perform multiple comparisons and analyses of TEG parameters to determine associations between *in vitro* whole blood viscoelastic properties. As a consequence of the small numbers and also due to the different time frames of the two cohorts, the pre-implementation and post-implementation groups did have characteristics that showed a statistical trend toward significance with respect to ECMO configuration (90 vs. 71% receiving VA-ECMO in the pre- vs. post-implementation cohorts, *p* = 0.10) and indication (29 vs. 47% primarily receiving ECMO support for primarily respiratory failure in the pre- vs. post-implementation cohorts, *p* = 0.15). We attempted to control for these confounding factors through our statistical approach. The four variables that remained in our two models (Table 4) were the only ones that reached the threshold *p*-value of 0.2. Notably, VA vs. VV configuration of ECMO therapy and respiratory vs. cardiac diagnoses as indication for ECMO support did not reach this statistical threshold. Others have reported that bleeding complications have a statistical trend toward higher occurrence in VA- vs. VV-ECMO (26) and that patients receiving ECMO support for cardiac vs. respiratory indications have a higher daily relative risk for bleeding (3). Both of these reports have notable limitations, chief among which is lack of a standardized approach to anticoagulation management in these cohorts. The lack of definitive data correlating hemorrhagic risk with either ECMO configuration or indication is thus reflected in the lack of distinction in recommended anticoagulation management based on these sets of variables by ELSO in either their General Guidelines (6) or in the most recent edition ELSO Red Book (1). A systematic review currently underway of anticoagulation management for ECMO has thus far found no evidence for using different ECMO anticoagulation strategies by either mode or circuit configuration (27).

The pre-implementation cohort data was collected retrospectively via chart review. Minor and intermediate hemorrhagic complications, defined as the presence of clinical bleeding resulting in either pharmacologic dose adjustment or application of hemostatic agents and/or pressure, may have thus been missed if bleeding was not specifically noted in the daily note or bedside charting. The definition of major hemorrhagic complication and the presence of ICH of any severity would have been readily identified during retrospective chart review. In contrast, for the prospective cohort, the bedside care team completed a daily standardized form with the hemorrhagic and thrombotic complication categories, as part of Guideline implementation to facilitate accurate documentation of bleeding and clotting occurrence and severity. The difference in documentation requirements between the two cohorts and the subsequent introduced biases could have led to fewer identified occurrences of minor or intermediate hemorrhagic complications in the pre-implementation cohort that were deemed unnecessary to document explicitly and likely resulted in under-appreciation for the frequency of these bleeding events in this group. Nevertheless, elimination of recall and information bias would be expected to lead to increased recognition of minor and intermediate severity bleeding events in the pre-implementation cohort, resulting in an even greater reduction in bleeding rates than we demonstrated.

Another important limitation is the lack of a uniform severity of illness score that applies to both neonatal and pediatric ICU patients. Consequently, we were not able to adjust our analysis for severity of illness. Further, although not statistically significant, the post-implementation cohort trended toward receiving VV-ECMO support more frequently than VA-ECMO support. Nevertheless, our pre- and post-implementation cohorts were well-matched for age, weight, and diagnostic category requiring ECMO support.

Additionally, since documentation of assigned clinical state was not uniformly performed, we were unable to assess compliance with Guideline recommendations. Therefore, we were unable to ascertain the degree of Guideline compliance. However, copies of the Guideline were placed at every ECMO patient's bedside and in the physician work areas throughout the duration of each ECMO run. Extensive and repeated education and training process targeted each ICU and was tailored to each professional group. The laboratory testing schedule was also built into the electronic ordering system. To provide peer support to the bedside clinical team, an informal “anticoagulation consult team” comprised of the stakeholders and developers of the Guideline provided real-time response to questions regarding Guideline content and use. These efforts all promoted frequent discussion both during daily bedside rounds and throughout the day. Through collaboration and open communication among the ICU interprofessional team and with guidance from the anticoagulation consult team, Guideline familiarity increased and the team became more facile with Guideline use (data not shown). These components of our implementation strategy likely mitigated unintended deviation from the Guideline recommendations.

## Conclusions

Life-threatening hemorrhagic or thrombotic complications present one of the highest risks for children receiving ECMO and, at least in part, appear related to imperfect existing strategies to balance anticoagulation risk and preservation of ECMO circuit performance. Implementation of a context-responsive anticoagulation and transfusion guideline in a single center is feasible and is associated with a decrease in major hemorrhagic and thrombotic complications as well as reduced RBC transfusion volume. Further investigations are necessary to identify which laboratory tests provide the clearest understanding of coagulation homeostasis and understand which aspects of the Guideline provide the most benefit and which offer no or negative impact, whether Guideline compliance correlates with outcome, and whether Guideline implementation is sustainable over time.

## Data Availability Statement

The raw data supporting the conclusions of this article will be made available by the authors, without undue reservation.

## Ethics Statement

The studies involving human participants were reviewed and approved by Washington University Institutional Review Board. Written informed consent from the participants' legal guardian/next of kin was not required to participate in this study in accordance with the national legislation and the institutional requirements.

## Author Contributions

JL, LB, AV, and PS contributed conception and design of the study. LB organized the database. MM, NO'C, and MS assisted in data collection and data entry. RC and MW performed the statistical analysis. JL and LB co-wrote the first draft of the manuscript. All authors contributed to manuscript revision, read, and approved the submitted version.

## Conflict of Interest

The authors declare that the research was conducted in the absence of any commercial or financial relationships that could be construed as a potential conflict of interest.

## Abbreviations

ECMO: extracorporeal membrane oxygenation
ACT: activated clotting time
aPTT: activated partial thromboplastin time
PT: prothrombin time
INR: international normalization ratio
ELSO: Extracorporeal Life Support Organization
TEG: thromboelastography
ATIII: antithrombin III
RRT: renal replacement therapy
eCPR: extracorporeal cardiopulmonary resuscitation
ICH: intracranial hemorrhage
RBC: red blood cell
FFP: fresh frozen plasma.
