# Supplementary material for: Context-Responsive Anticoagulation Reduces Complications in Pediatric Extracorporeal Membrane Oxygenation
Source: Front Cardiovasc Med. 2021 Jun 10;8:637106. doi: 10.3389/fcvm.2021.637106 (PMC8224528; doi:10.3389/fcvm.2021.637106)
Supplement: Supplementary file 1 [file Data_Sheet_1.PDF]

ECMO Transfusion and Hemostasis Guidelines

1) Pre-ECMO

Obtain the following to assist with post-ECMO anticoagulation:

a. Thorough patient and family history regarding risk for congenital hypo or hypercoagulable disorders.

b. Within 4 hours prior to ECMO placement, obtain the following coagulation testing:
   i. CBC for platelet count
   ii. PT/INR/PTT, fibrinogen
   iii. AT-III
   iv. anti-Xa (if pt on Heparin or LMWH, or immediately post CPB)
   v. baseline ACT
   vi. TEG (citrate, heparinase, and platelet mapping).

c. If abnormalities are present in these initial labs unexplained by the clinical situation and there is a history suggestive of a hypo- or hypercoagulation disorder, consult hematology to evaluate for a possible congenital coagulation disorder.

2) Initiation of ECMO

a. Heparin bolus of 50-100 units/kg IV.

b. Within 30 minutes, start heparin infusion at 28 units/kg/hr for patients ≤ 1 year old or 20 units/kg/hr for patients > 1 year old.

c. Heparin bolus and infusion may not be initiated if severe bleeding is present.

3) During ECMO

Three pathways apply to (1) patient without bleeding or clotting, (2) patient with bleeding, and (3) patient with clotting. These pathways guide hemostatic testing, coagulation management, and non-RBC transfusion. Deviation from these guidelines should be performed only with attending involvement and approval.

a. <u>Summary of Figures and Tables</u>
   i. Figure 1: ECMO Anticoagulation—No Bleeding / No Clotting
   ii. Figure 2: ECMO Anticoagulation—Bleeding
   iii. Figure 3: ECMO Anticoagulation—Clotting
   iv. Table 1: Recommended starting ACT goals
   v. Table 2: Risk factors for spontaneous intracranial hemorrhage
   vi. Table 3: Heparin titration algorithms—ACT and anti-XA based
   vii. Table 4: Bleeding and clotting complication categories

b. <u>RBC transfusion indications</u> **[Dose: 10-15ml/kg, max 2 units]**
   Per Blood Bank policy all RBCs transfused to patients on ECMO should be less than 7 days of storage. If the storage age is > 7 days discuss with the blood bank the use of volume reduced or washed RBCs if appropriate.

   i. Hemorrhagic Shock (anaerobic state)

   ii. Shock that cannot be reversed by other means of increasing oxygen delivery to include:
      1. Increasing pump flow

2. Increasing afterload reduction
3. Reducing patient oxygen consumption

iii. No evidence of shock and Hb < 10g/dl if clinically indicated.

iv. Consider 15ml/kg RBC transfusion at 24 hours post ECMO initiation in all children < 6kg prior to the development of anemia.

c. FFP transfusion indications **[Dose: 10-15ml/kg, max 2 units]**

i. Life-threatening bleeding: Per massive transfusion protocol.

ii. Significant active bleeding associated with one of the following:
1. INR > 1.7
2. Heparinase TEG-R > 12
3. In bleeding patients with above INR and TEG-R criteria and ACT > 180 seconds, reduce heparin infusion if dose is > 20 U/kg/hr. Magnitude of reduction should be based upon severity of bleeding and clinical risk of adverse thrombotic events in the patient being treated (see Fig 2 and Tab 3).

iii. For non-bleeding patients:
1. INR > 2.0
2. Heparinase TEG-R > 15
3. In non-bleeding patients with above INR and TEG-R criteria, consider reducing heparin infusion if dose is > 20 U/kg/hr. Magnitude of reduction should be based upon severity of bleeding and clinical risk of adverse thrombotic events in the patient being treated.

d. Platelet transfusion indications **[Dose: 10-15ml/kg, max 2 units]**

i. Life-threatening bleeding: Per Massive transfusion protocol

ii. Non-bleeding patient:
1. Platelet < 100,000 for patients at high risk for intracranial hemorrhage (see Tab 2).
2. Platelet < 50,000 if not at high risk of ICH.
3. Heparinase TEG G value < 4 $dynes/sec^2$

iii. Actively bleeding patient:
1. Platelet count < 100,000.
2. Heparinase TEG G value < 6 $dynes/sec^2$

e. Cryoprecipitate indications **[Dose: 1 unit / 10kg wt, max 10 units]**

i. Life-threatening bleeding: Per massive transfusion protocol

ii. Significant bleeding associated with reduced fibrinogen function defined as:
1. Fibrinogen < 200
2. Heparinase TEG K-time > 3min

iii. If no clinical bleeding
1. Fibrinogen < 100
2. Heparinase TEG K time > 5 min

f. Anti-fibrinolytic agents: Amicar or Tranexemic acid (Attending physican must approve)

i. Significant bleeding associated with hyperfibrinolysis: heparinase TEG LY30 > 5%

ii. Empirically for major surgical procedures at the discretion of the treating physicians

g. <u>AT-III indications</u>

   i. Reduced heparin effect on citrate TEG vs heparinase TEG comparison, defined as delta-R value difference of < 0.3 min.

   ii. Anti-Xa < 0.4.

   iii. Clinically significant thrombotic events.

   iv. Measured AT-III activity 20 minutes after bolus dosing and then at least q12 whether using bolus dosing or if using a continuous infusion.

   v. AT-III bolus dose = [(goal – measured activity) / goal activity] x total plasma volume

   Total plasma volume = estimated patient + circuit plasma volume
   = [(0.065 x wt x 1000ml/kg) + circuit vol] x (1-Hct)

   Example: wt=10kg, Hct=30%, AT-III activity=40% (goal=70%), circuit total volume=250ml

   AT-III dose = [(0.7 – 0.4) / 0.7] x [(0.065 x 10kg x 1000ml/kg0 + 250] x (1 – 0.3)
   = 0.3 / 0.7 x 900ml x 0.7
   = 270 units

h. <u>Anticoagulation</u>
After Initiation of ECMO anticoagulation management will consist of a two-tiered approach:

   i. **Tier 1 Therapy** (ACT-guided heparin infusion titration).

      1. Set ACT goal based on risk of bleeding or thrombosis (see Tab 1).

      2. HepCon is replaced with anti-Xa activity. Low, normal, and high HepCon results are equivalent to anti-Xa of <0.4, 0.4-0.8, > 0.8, respectively.

      3. If ACT results are discordant with anti-Xa and/or TEG results or if significant bleeding/thrombotic events begin to occur, then move to **Tier 2 Therapy**.

   ii. **Tier 2 Therapy** (anti-Xa guided heparin infusion titration).

      1. Follow q8 hr anti-Xa with goal Anti-Xa = 0.4 – 0.8.

      2. Repeat anti-Xa value 4 hours after each heparin infusion change.

      3. If anti-Xa below range and heparin dose ≥ 50U/kg/hr, check AT-III activity.

      4. Goal TEG heparinase G value of 6-12 dynes/sec^2

         a. Consider antiplatelet agents if G > 12 dynes/sec^2 and there is concern or increased risk of adverse thrombotic events specific to the patient. Dipyridimole is the first line choice with aspirin as the second. If either are initiated goal is for inhibition at ADP or AA receptor between 60-80%.

         b. Discontinue antiplatelet agents if G < 6 dynes/sec^2

i. <u>Lab schedule</u>

   i. Immediately following ECMO initiation, send ACT and then q1 hour until heparin dose unchanged for 6 hours and then q2 hours.

ii. For non-bleeding/non-clotting patients (Tier 1 therapy):

1. q1 hr ACT until heparin dose unchanged for 6 hours, then q2 hr

2. q8 hr CBC

3. q12 hr x 6 then qAM:
    a. PT/INR/PTT, fibrinogen
    b. anti-Xa, AT-III
    c. TEG (citrate, heparinase, platelet mapping)

iii. For bleeding or clotting patients (Tier 2 therapy):

1. q4 hr ACT (for recording purposes only)

2. q8hr anti-Xa (or 4 hours after each heparin change)

3. q8hr CBC

4. q8hr x 6 then qAM (retime to coincide with anti-Xa levels following heparin changes)
    a. PT/INR/PTT, fibrinogen
    b. AT-III
    c. TEG (citrate, heparinase, platelet mapping)

iv. The above set of labs will be evaluated in the clinical context of the patient and the degree of clotting present within the ECMO circuit.

j. Moving between pathways
Moving between pathways is dependent on appropriate and timely recognition and categorization of hemorrhagic and thromboembolic complications (Table 4).

i. All patients begin on the "No Bleeding / No Clotting" pathway at the start of their ECMO course.

ii. "Bleeding" pathway:
1. Begin pathway for hemorrhagic major (H1) or intermediate (H2) complication.
2. Return to "No Bleeding / No Clotting" pathway after:
    a. 48 hours of no bleeding
    b. 48 hours of no transfusion requirements of platelets, FFP, cryoprecipitate

iii. "Clotting" pathway:
1. Begin pathway for patient thrombotic/embolic major or circuit thrombotic major complications. Remain in "Clotting" pathway for the remaining duration of the ECMO run.
2. If bleeding develops meeting H1 or H2 criteria, transition to "Bleeding" pathway. Return to "Clotting" pathway after:
    a. 48 hours of no bleeding
    b. 48 hours of no transfusion requirements of platelets, FFP, cryoprecipitate

4) Post ECMO surveillance

a. Doppler exam of great vessels within 48 hours.

b. Head ultrasound for infants within 48 hours.

c. Brain MRI within 7 days. For children who may require sedation/intubation for the MRI the study will be scheduled prior to extubation after decanulation. Extubation should not be significantly delayed to complete the MRI study of the brain.

Appendix: ECMO transfusion and anticoagulation guidelines

CCM Coagulation Team

CICU	S.Baltagi, A.Gazit
PICU	J.Lin, P.Spinella
NICU	A.Vogel

AT-III Dose Calculation

Bolus Dose = [(goal - measured activity / goal activity] x total plasma volume (in ml)
total plasma vol (ml) = estimated patient + circuit plasma vol
= [(0.065 x wt x 1000ml/kg) + circuit volume] x (1-Hct)

Example: 10kg pt, Hct=30%, AT-III activity=40%, goal activity=70%, circuit total vol=250ml

Dose = [(0.7 - 0.4) / 0.7] x [(0.065 x 10kg x 1000ml/kg) + 250ml] x (1 - 0.3)
= 0.3 / 0.7 x 900ml x 0.7
= 270 units

Table 1: Suggested starting anti-Xa goals
Confirm all anti-Xa goals with CCM / PedSurg / CTSurg attending prior to initiation.

ECMO for / in:	Default anti-Xa goal
Diaphragmatic Hernia, pre-op	0.3-0.7
Diaphragmatic Hernia, post-op	0.3-0.7
Respiratory Failure	0.3-0.7
Refractory Septic Shock	0.3-0.7
Post cardiac arrest	0.3-0.5
Cardiac Failure, non-surgical (e.g. myocarditis)	0.3-0.7
Cardiac Failure, surgical (e.g. post-op LCOS)	0.3-0.5
Trauma, bleeding	0.3-0.5
Trauma, not bleeding	0.3-0.7
Post-surgical patient, bleeding	0.3-0.5
Post-surgical patient (<72 hours post-op), not bleeding	0.3-0.7
Post-surgical patient (>72 hours post-op), not bleeding	0.3-0.7
Neurosurgical post-op patient	discuss with NSGY

Table 2: Increased risk of spontaneous intracranial hemorrhage.
If present, default anti-Xa goal should be decreased to 0.3-0.5.

- age < 3 months
- s/p cardiac arrest (ischemia / reperfusion)
- pre-existing intracranial vascular anomaly (e.g. AVM, moya-moya)
- pre-existing intracranial mass
- h/o recent ischemic stroke (risk of hemorrhage conversion)

Table 3a: ACT-based Heparin Titration Algorithm
For use ONLY when heparin changes are based on ACT alone.
Notify primary attending before any heparin boluses or infusion holds.
Repeat ACT q1 hour per protocol but limit infusion rate changes to q4 hours.
Heparin dose changes more frequent than q4 hours must be discussed with attending.

ACT	Infusion Dose adjustment instructions	Next heparin adjustment
>40% below lower limit	Bolus 20 units/kg, then increase infusion 20%.	4 hours
>20% below lower limit	Bolus 10 units/kg, then increase infusion 15%.	4 hours
>10% below lower limit	Increase infusion 10%.	4 hours
<10% below lower limit	No change unless clotting. If clotting, increase infusion 10%.	1 hour if no change. 4 hours if changed.
In range	No change.	
<10% above upper limit	No change unless bleeding. If bleeding, decrease infusion 10%.	1 hour if no change. 4 hours if changed.
>10% above upper limit	Decrease infusion 10%.	4 hours
>20% above upper limit	Hold infusion for 30 minutes, then decrease infusion 10%.	4 hours
>50% above upper limit	Hold infusion for 60 minutes, then decrease infusion 15%.	4 hours
>75% above upper limit	Hold infusion for 90 minutes, then decrease infusion 20%.	4 hours

Table 3b: anti Xa-based Heparin Titration Algorithm
For use ONLY when heparin changes are based on anti-Xa alone.
Notify primary attending before any heparin boluses or infusion holds.
Repeat anti-Xa level 4 hours after any infusion change, re-adjust infusion accordingly.
Heparin dose changes more frequent than q4 hours must be discussed with attending.

anti-Xa	Infusion Dose adjustment instructions	Next heparin adjustment
<0.20	Increase infusion 20%.	4 hours
0.20-0.39	Increase infusion 10%.	4 hours
0.40-0.80	No change.	
0.80-0.89	Decrease infusion 10%.	4 hours
0.90-1.09	Hold infusion for 30 minutes, then decrease infusion 10%.	4 hours
1.10-1.40	Hold infusion for 60 minutes, then decrease infusion 15%.	4 hours
>1.40	Hold infusion for 60 minutes, then decrease infusion 20%.	4 hours

Table 4: Complications Categories and Examples

**Hemorrhagic Major (H1)**	1. Bleeding requiring surgical intervention for bleeding<br>-wound exploration<br>-laparotomy<br>-thoracotomy
	2. Bleeding requiring discontinuation of ECLS and decannulation<br>- Grade IV IVH
	3. Bleeding causing death
	4. Overt clinical bleeding requiring > 20 ml/kg/day PRBC transfusion
**Hemorrhage Intermediate (H2)**	1. Neurologic complication requiring pharmacologic* adjustments but not requiring decannulation<br>- new grade I IVH
	2. Other clinically overt internal or external bleeding complication requiring pharmacologic and additional interventions.<br>-pulmonary bleeding requiring heparin adjustment and a change in ventilator parameters<br>-gastrointestinal bleeding requiring heparin adjustment and holding enteral feeds<br>-urologic bleeding requiring heparin adjustment and removing the Foley catheter
**Hemorrhage Minor (H3)**	1. Clinical bleeding requiring > 10 ml/kg/day PRBC transfusion
	2. Bleeding requiring ONLY pharmacologic intervention<br>- Heparin dose adjustment
	3. Bleeding requiring non-surgical intervention<br>- application of topical hemostatic agent to incision
**Patient Thrombotic Major (PTM)**	1. Patient thrombosis requiring discontinuation of ECLS
	2. Patient thrombosis resulting in death
**Patient Embolic Major (PEM)**	1. Embolic event requiring discontinuation of ECLS
	2. Embolic event resulting in death
**Circuit Thrombotic Major (CTM)**	1. Acute circuit thrombosis requiring component or circuit change<br>Note: would not include natural progression of a circuit on a long run as a complication but would record the circuit change but not as a thrombotic complication
**Thromboembolic Minor (TM)**	1. Clinically overt patient or circuit thromboembolic event requiring pharmacologic intervention<br>-increasing heparin dose

* would include heparin adjustments, transfusing coagulation factor such as FFP, fibrinogen, etc.

ECMO Anticoagulation:
No Bleeding / No Clotting

20130813

v. 20140512

- Set target platelet and ACT goals. See Table 1 for suggested starting ACT goals*.
- Identify patients with increased risk of spontaneous intracranial hemorrhage (Table 2).
- Follow Tier 1 lab schedule. Indicated labs are in addition to standard schedule.
- Start heparin at 20 units/kg/hr and adjust per **ACT**-titration algorithm (Table 3a).

Tier 1 Coagulation Lab Schedule

- q1hr ACT until no heparin gtt change for >6hr. Then q2 hr ACT.
- q8hr CBC
- q12 x **6** then qAM:
  PT/INR/PTT/fibrinogen
  anti-Xa, AT-III
  TEG (citrate, heparinase, platelet mapping)

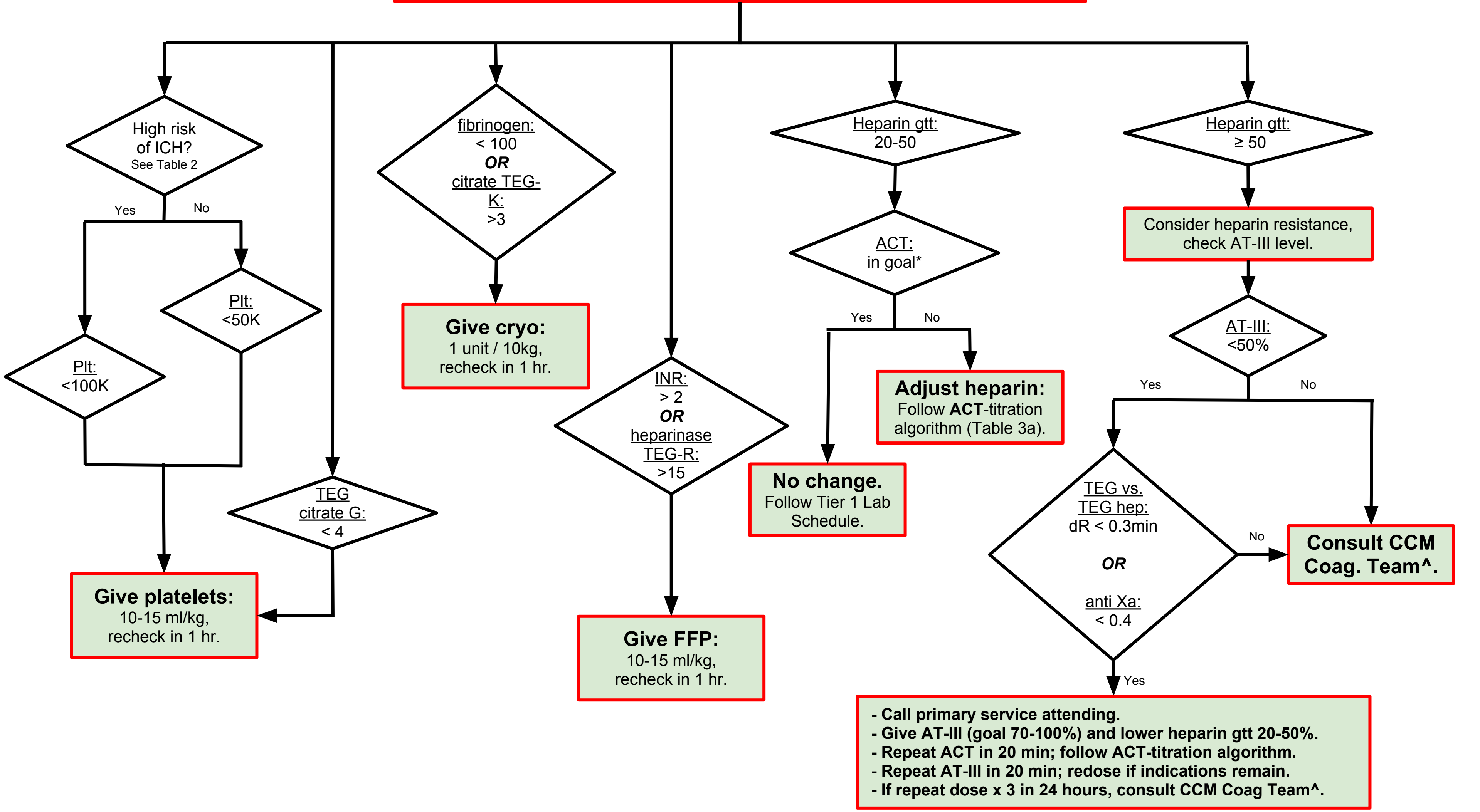

AT-III Dose Calculation

AT-III dose = [(goal - measured activity) / goal activity] x total plasma volume (ml)
total plasma vol (ml) = estimated patient + circuit plasma vol
= [(0.065 x wt x 1000ml/kg) + circuit vol] x (1-Hct)

e.g.: 10kg pt, Hct=30%, AT-III activity=40%, goal activity=70%, circuit total vol=250ml
AT-III dose = [(0.7 - 0.4) / 0.7] x [(0.065 x 10kg x 1000ml/kg) + 250ml] x (1 - 0.3)
= 0.3 / 0.7 x 900ml x 0.7
= 270 units

* Confirm ACT goal with attending.
^ CCM Coag. Team:
CICU: S.Baltagi, A.Gazit
PICU: J.Lin, P.Spinella
NICU: A.Vogel

ECMO Anticoagulation: Bleeding

20131218

v. 20140512

- Primary physician team **MUST** initiate all steps in this pathway.
- Categorize bleeding severity (Table 4); call surgeon if H1 or H2; rule out surgical bleeding.
- If bleeding does not stop with **ACT**- titration algorithm, **stop ACT-based titration**. Adjust heparin using **anti-Xa** algorithm. Start Tier 2 guided anticoagulation and lab schedule.
- Consider holding heparin for severe bleeding; **activate MTP for life threatening bleeding.**

Tier 2 Coagulation Lab Schedule
- q4hr ACT (for recording purposes only).
- q8hr anti-Xa (or 4 hours after each heparin change)
- q8hr CBC
- q8 x **6** then qAM (retime to coincide with each anti-Xa levels following heparin changes)
  PT/INR/PTT/fibrinogen, AT-III
  TEG (citrate, heparinase, platelet mapping)

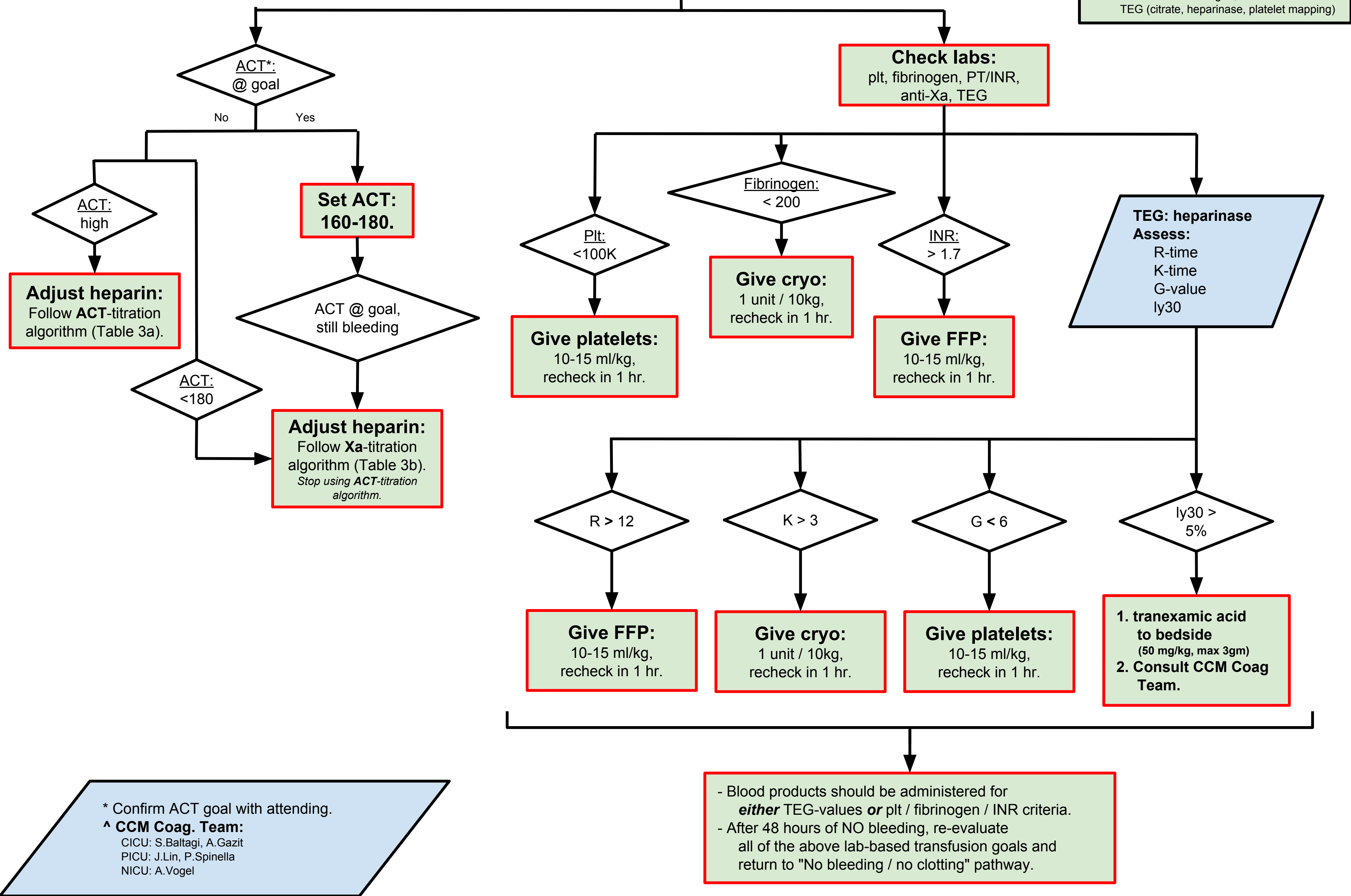

ECMO Anticoagulation: Clotting

20130813

v. 20140512

Tier 2 Coagulation Lab Schedule
- q4hr ACT (for recording purposes only).
- q8hr anti-Xa (or 4 hours after each heparin change)
- q8hr CBC
- q8 x **6** then qAM (retime to coincide with each anti-Xa levels following heparin changes)
  PT/INR/PTT/fibrinogen, AT-III
  TEG (citrate, heparinase, platelet mapping)

Check heparin drip concentration / dosage / administration technique.
Categorize clotting severity (See Table 4).
Discuss need for circuit / oxygenator change.
Assess for:
- heparin resistance (anti-Xa level)
- new infectious / inflammatory source
- increased platelet activity
- mechanical causes for circuit clotting
- platelet consumption vs. heparin induced thrombocytopenia (HIT)

Start Tier 2 guided anticoagulation and lab schedule.
Adjust heparin using **anti-Xa** algorithm; **stop using ACT algorithm**.

Heparin gtt: ≥ 50

- No → Stop **ACT**-based titration. Adjust heparin using **Xa**-titration algorithm.
  - **anti-Xa > 0.8** → **- Evaluate for mechanical causes for circuit clotting. - Consult CCM Coag Team^.**
  - **anti-Xa < 0.4** → **Adjust heparin:** Follow **Xa**-titration algorithm (Table 2b). **Consult CCM Coag Team if AT-III <50%.**
- Yes → Consider heparin resistance, check AT-III level.
  - **AT-III: <50%**
    - No → **Consult CCM Coag. Team.^**
    - Yes → **TEG vs. TEG hep:** dR < 0.3min ***OR*** **anti Xa:** < 0.4 ***OR*** major thrombotic event (Table 4)
      - No → **Consult CCM Coag. Team.^**
      - Yes →
        - **Call primary service attending.**
        - **Give AT-III (goal 70-100%) and lower heparin gtt 20-50%.**
        - **Repeat ACT in 20 min; follow ACT-titration algorithm.**
        - **Repeat AT-III in 20 min; redose if indications remain.**
        - **If repeat dose x 3 in 24 hours, consult CCM Coag Team^.**

heparinase TEG G: >12 → **Consider anti-platelet agents.** **Discuss with CCM Coag Team^ and Cannulating ECMO Surgeon.**

plt: < 100 →

Assess circuit consumption.
- clots isolated to circuit

Consider DIC.
- send PT/INR/PTT, fibrinogen, D-dimer, TEG (citrate + heparinase), CBC
- **Consult CCM Coag Team^.**

Consider HIT if:
- thromboembolic disease in patient's small arterial / venous vessels
- acute, precipitous platelet drop

If HIT is possible:
- Send HIT antibody panel.
- **Consult CCM Coag Team^.**

^ CCM Coag. Team:
CICU: S.Baltagi, A.Gazit
PICU: J.Lin, P.Spinella
NICU: A.Vogel

AT-III Dose Calculation

AT-III dose = [(goal - measured activity) / goal activity] x total plasma volume (ml)
total plasma vol (ml) = estimated patient + circuit plasma vol
= [(0.065 x wt x 1000ml/kg) + circuit vol] x (1-Hct)

e.g.: 10kg pt, Hct=30%, AT-III activity=40%, goal activity=70%, circuit total vol=250ml
AT-III dose = [(0.7 - 0.4) / 0.7] x [(0.065 x 10kg x 1000ml/kg) + 250ml] x (1 - 0.3)
= 0.3 / 0.7 x 900ml x 0.7
= 270 units
